# New Method of Treatment of Febrile Articular Rheumatism

**Published:** 1876-01

**Authors:** 


A New Method of Treatment of Febrile Articular Rheumatism—(*Centralblatt fur Chirurgie, from Deut. Zeitschrift fur Prakt. Med.*)—The treatment consisted in the hypodermic use of a solution of carbolic acid, and has been tried for a period of two and a half years. One or two joints were placed under treatment simultaneously, by making one or two injections of a solution of carbolic acid, of a strength of two per cent., at the most painful points. No irritation usually resulted, and if there was any it was of but slight intensity. The acid seemed to act almost as an anæsthetic, for in a period varying in length from half an hour to several hours all pain ceased, the patient fell asleep, the swelling of the joint diminished, and but a slight amount of stiffness remained. After the lapse of a few days all symptoms of a rheumatic character vanished from the joint, and in but few of the cases did any relapse occur. The action of the carbolic acid was purely local, and influenced the fever only so far as it was due to the local inflammation. Quinine was also used in gramme doses; and Kunz thinks that by combining these two modes of treatment it is possible to shorten the course of the disease to a considerable extent.—*Phil. Med Times.*

